# Vascular Complications of Intercavernous Sinuses during Transsphenoidal Surgery: An Anatomical Analysis Based on Autopsy and Magnetic Resonance Venography

**DOI:** 10.1371/journal.pone.0144771

**Published:** 2015-12-10

**Authors:** Xuefei Deng, Shijun Chen, Ya Bai, Wen Song, Yongchao Chen, Dongxue Li, Hui Han, Bin Liu

**Affiliations:** 1 Department of Anatomy, Anhui Medical University, Hefei, Anhui, China; 2 Department of Radiology, The First Affiliated Hospital of Anhui Medical University, Hefei, Anhui, China; 3 Ultrasonic Center, The 105th Hospital of PLA, Hefei, Anhui, China; 4 Department of Neurosurgery, Anhui Provincial Hospital, Hefei, Anhui, China; Shenzhen institutes of advanced technology, CHINA

## Abstract

**Purpose:**

Vascular complications induced by intercavernous sinus injury during dural opening in the transsphenoidal surgery may contribute to incomplete tumour resections. Preoperative neuro-imaging is of crucial importance in planning surgical approach. The aim of this study is to correlate the microanatomy of intercavernous sinuses with its contrast-enhanced magnetic resonance venography (CE-MRV).

**Methods:**

Eighteen human adult cadavers and 24 patients were examined based on autopsy and CE-MRV. Through dissection of the cadavers and CE-MRV, the location, shape, number, diameter and type of intercavernous sinuses were measured and compared.

**Results:**

Different intercavernous sinuses were identified by their location and shape in all the cadavers and CE-MRV. Compared to the cadavers, CE-MRV revealed 37% of the anterior intercavernous sinus, 48% of the inferior intercavernous sinus, 30% of the posterior intercavernous sinus, 30% of the dorsum sellae sinus and 100% of the basilar sinus. The smaller intercavernous sinuses were not seen in the neuro-images. According to the presence of the anterior and inferior intercavernous sinus, four types of the intercavernous sinuses were identified in cadavers and CE-MRV, and the corresponding operative space in the transsphenoidal surgical approach was implemented.

**Conclusion:**

The morphology and classification of the cavernous sinus can be identified by CE-MRV, especially for the larger vessels, which cause bleeding more easily. Therefore, CE-MRV provides a reliable measure for individualized preoperative planning during transsphenoidal surgery.

## Introduction

The intercavernous sinuses are the venous interconnections between the bilateral cavernous sinuses in the dura mater around the pineal gland. To date, strong efforts have been made to reveal the anatomical variations of this special venous structure [1–5]. According to the different locations of the intercavernous sinuses surrounding the pineal gland, an anatomist can divide them into the anterior intercavernous sinus, inferior intercavernous sinus, posterior intercavernous sinus, dorsum sellae sinus, and basilar sinus [3].

Transsphenoidal surgery has been well established as an effective primary treatment for tumours of the sellar region due to its minimal invasiveness, low morbidity and excellent surgical outcome [6–9]. During the dural opening, the intercavernous sinuses (especially the anterior and inferior intercavernous sinuses) are prone to bleeding under inappropriate manipulation, and may lead to poor visualization, inadequate exposure, and incomplete tumour resection [10–13]. Imaging analysis of the sellar region is of high importance in the planning, execution and outcome of the transsphenoidal surgery [14–16]. Therefore, preoperative neuro-radiological information on the intercavernous sinus may also be of crucial importance for avoiding bleeding in transsphenoidal surgery.

Although the venography of intercavernous sinuses was used to diagnose the pituitary adenomas [17, 18], few studies to date have attempted to evaluate the diagnosis value of the intercavernous sinus itself with different neuro-imagings [5]. Data from human cadavers were used as a reference to evaluate the specificity of the imaging modality in visualizing intracranial vascular structures [19–22]. Magnetic resonance imaging (MRI) has become increasingly popular as a non-invasive means of evaluating the intracranial system. Therefore, the aim of this study is to correlate the microanatomy of intercavernous sinuses with its MRI images in order to provide preoperative information for transsphenoidal surgery.

## Materials and Methods

Eighteen adult cadavers (6 females, 12 males; age range, 20–63 years; mean age, 46 years) and 24 patients (11 females, 13 males; age range, 24–72 years; mean age, 42 years) were examined in this study from April 2014 to May 2015. The study was approved by the Ethics Committee of Anhui Medical University (Hefei, China, 2012238). The cadavers used by the Department of Anatomy of our institution for research and educational purposes were assigned to this project with permission given by their next-of-kin(s). All the participants provided their written informed consent to participate in this study.

To obtain true sensitivity, MRI is performed in order to compare with the image analysis of the cadavers pertaining to the same subjects. In this study, we evaluated the MRI sensitivity by the comparing of the data collected separately from MRI and the adult cadavers, mentioned by Kilic et al [19] and Han et al [20–22]. Each neuro-image was reviewed by two neuro-radiologists who provided the image from our research team and a neurosurgeon, who had more than three years of experience in transsphenoidal surgery.

### Autopsy analysis

The cadavers were fixed with a 10% formalin solution via the right femoral artery perfusion within 36 hours after death. The internal jugular veins were perfused with blue-coloured latex. The specimens were sawed along their median sagittal plane. The characteristics based on the location, shape, number, diameter and type of the intercavernous sinuses were measured and recorded.

### Contrast-enhanced magnetic resonance venography analysis

The indications for CE-MRV were suspicion of an intracranial lesion (eleven patients), and evaluation of lacunar cerebral infarction (thirteen patients). The patients who were diagnosed with intracranial lesion or cerebral venous diseases were excluded from our study. The coronary images were obtained using a standard head coil in a 3.0T scanner (General Electric, Milwaukee, USA). Paramagnetic contrast medium (Gd-DTPA, 20 ml of 0.1 mmol/kg solution; Amersham Health, Princeton, NJ, USA) was injected into a cubital vein at a rate of 2–3 ml/second. Sections of 1.4 mm thickness were obtained in the coronal plane using the following parameters: 3.2/1.3 (TR/TE), 60° flip angle, 240×240 mm field of view. All the images were transferred to the Advantage Windows 3D workstation for reconstruction. The 3D MRA images were obtained by the maximum pixel intensity projection method using Ph8/9 (SUB: Cor-Tricks-Asset). The mentioned characteristics of the intercavernous sinuses were measured and compared with the data from the cadavers.

### Statistical analysis

The data were analyzed using the Data Analysis Tool Kit included in Microsoft Excel and tested by the chi-square test and the student’s t test (for independent samples) analysis. A *P* value of < 0.05 was considered to indicate a significant difference.

## Results

### Location and shape of intercavernous sinus

**Fig 1:**
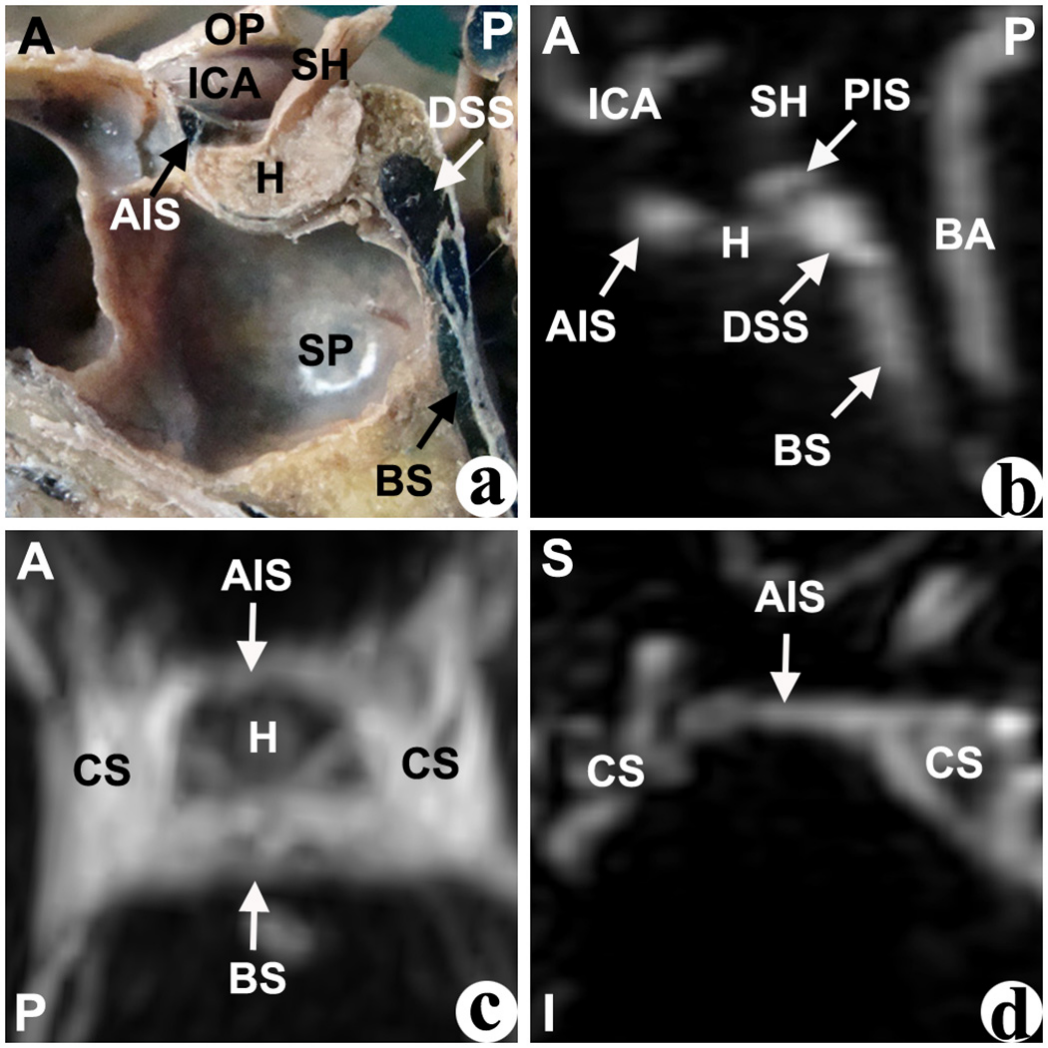
Anterior intercavernous sinus but not inferior intercavernous sinus was found in the cadaver (a) or CE-MRI image (b: sagittal, c: horizontal, d: coronal). AIS: anterior intercavernous sinus; BA: basilar artery; BS: basilar sinus; CS: cavernous sinus; DSS: dorsum sellae sinus; H: hypophysis; ICA: internal carotid artery; OP: optic nerve; SH: stalk of hypophsis; SP: sphenoid sinus. Direction: A-superior; P-posterior; S-superior; I-inferior.

**Fig 2:**
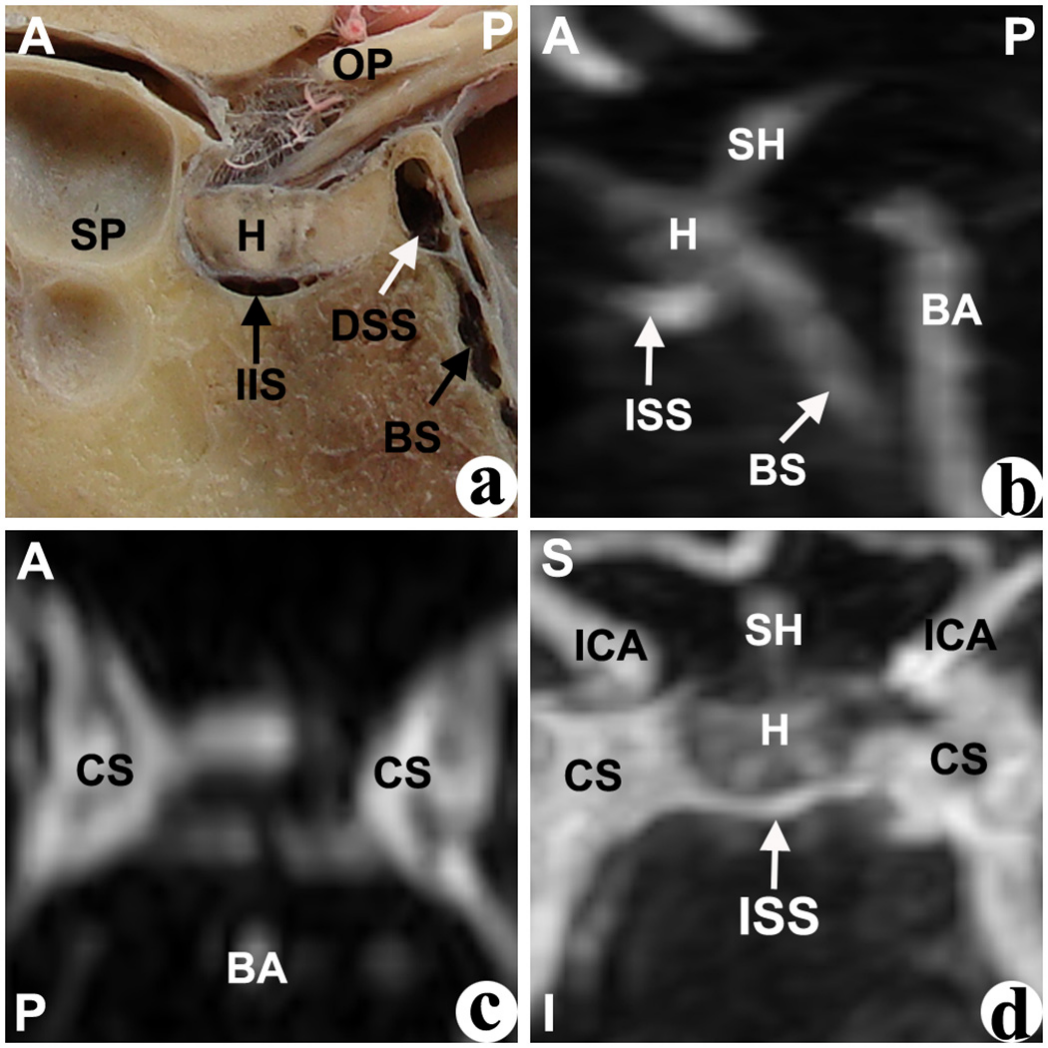
Inferior intercavernous sinus but not anterior intercavernous sinus was found in the cadaver (a) or CE-MRI image (b: sagittal, c: horizontal, d: coronal). BA: basilar artery; BS: basilar sinus; CS: cavernous sinus; DSS: dorsum sellae sinus; H: hypophysis; ICA: internal carotid artery; IIS: inferior intercavernous sinus; OP: optic nerve; SH: stalk of hypophsis; SP: sphenoid sinus. Direction: A-superior; P-posterior; S-superior; I-inferior.

**Fig 3:**
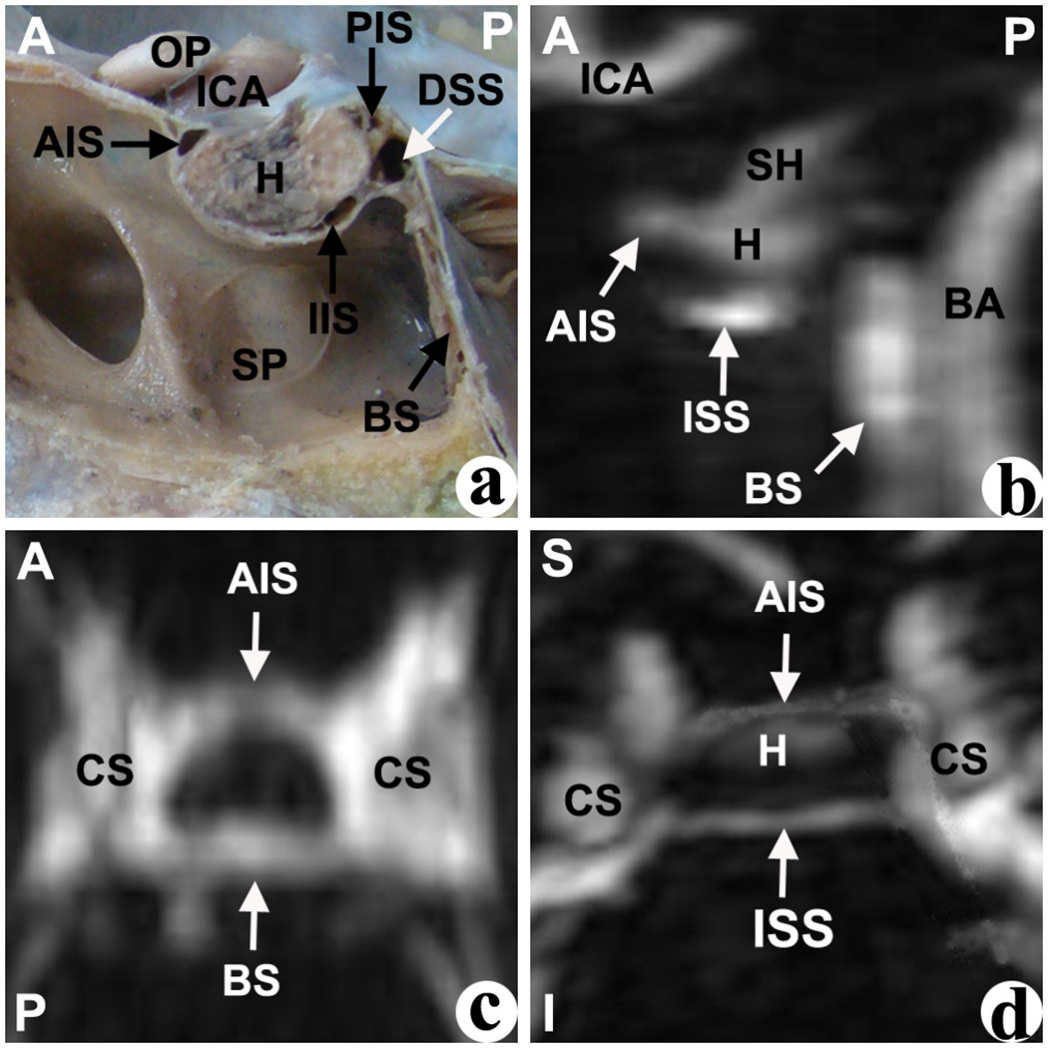
Both the anterior intercavernous sinus and the inferior intercavernous sinus were found in the cadaver (a) or CE-MRI image (b: sagittal, c: horizontal, d: coronal). AIS: anterior intercavernous sinus; BA: basilar artery; BS: basilar sinus; CS: cavernous sinus; DSS: dorsum sellae sinus; H: hypophysis; ICA: internal carotid artery; IIS: inferior intercavernous sinus; OP: optic nerve; SH: stalk of hypophsis; SP: sphenoid sinus. Direction: A-superior; P-posterior; S-superior; I-inferior.

**Fig 4:**
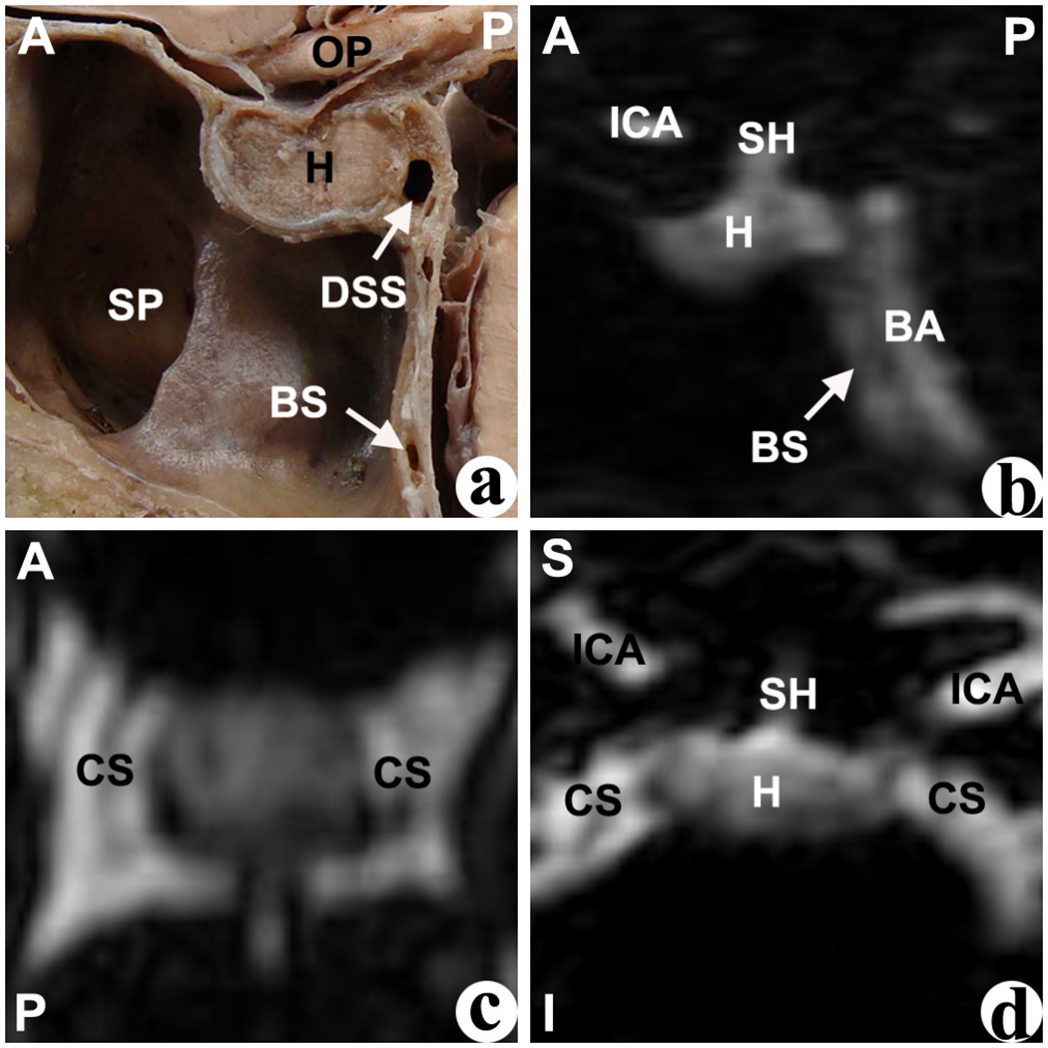
Neither the anterior intercavernous sinus nor the inferior intercavernous sinus were found in the cadaver (a) or CE-MRI image (b: sagittal, c: horizontal, d: coronal). BA: basilar artery; BS: basilar sinus; CS: cavernous sinus; DSS: dorsum sellae sinus; H: hypophysis; ICA: internal carotid artery; OP: optic nerve; SH: stalk of hypophsis; SP: sphenoid sinus. Direction: A-superior; P-posterior; S-superior; I-inferior.

In the median sagittal plane of the cadavers’ dissections, different cavernous sinuses were identified by their shape and their relative location to the pituitary fossa. The anterior intercavernous sinus (AIS) was located at the anterior-superior edge of the pituitary fossa, and was mostly presented as an approximate triangular shape (Fig 1). The inferior intercavernous sinus (IIS), which was located at the anterior-inferior edge of the junction of the anterior and posterior pituitary gland, was mostly presented as a crescentiform or oval shape (Fig 2). The posterior intercavernous sinus (PIS), whichi was located at the posterior-superior of pituitary gland, was typically presented with an oval shape (Fig 3). Furthermore, the dorsum sellae sinus (DSS) at the superior of the dorsum sellae had an approximate spherical shape, and the basilar sinus (BS) at the posterior of the clivus was mostly composed of a number of cord- or septo-shaped cavities (Figs 1–4).

In the median sagittal plane of the anatomical structure based on CE-MRV, the entire gland enhanced homogeneously, and the intercavernous sinus had a higher intensity surrounding the gland. Similar to the cadavers, different intercavernous sinuses were identified by their different locations around the gland (Figs 1–4). The inferior part of the DSS was connected with the BS, and sometimes there are no limpid boundaries between them (Figs 2–4). The AIS clearly demonstrated in the transverse plane, and the IIS was typically seen in the coronal plane most of the time. In the multi-planar reconstruction imaging, the anatomical revelations based on the transverse and coronal plane were compacted onto an image in the sagittal plane (Figs 1–4).

### Sensitivity of contrast-enhanced magnetic resonance venography

**Fig 5:**
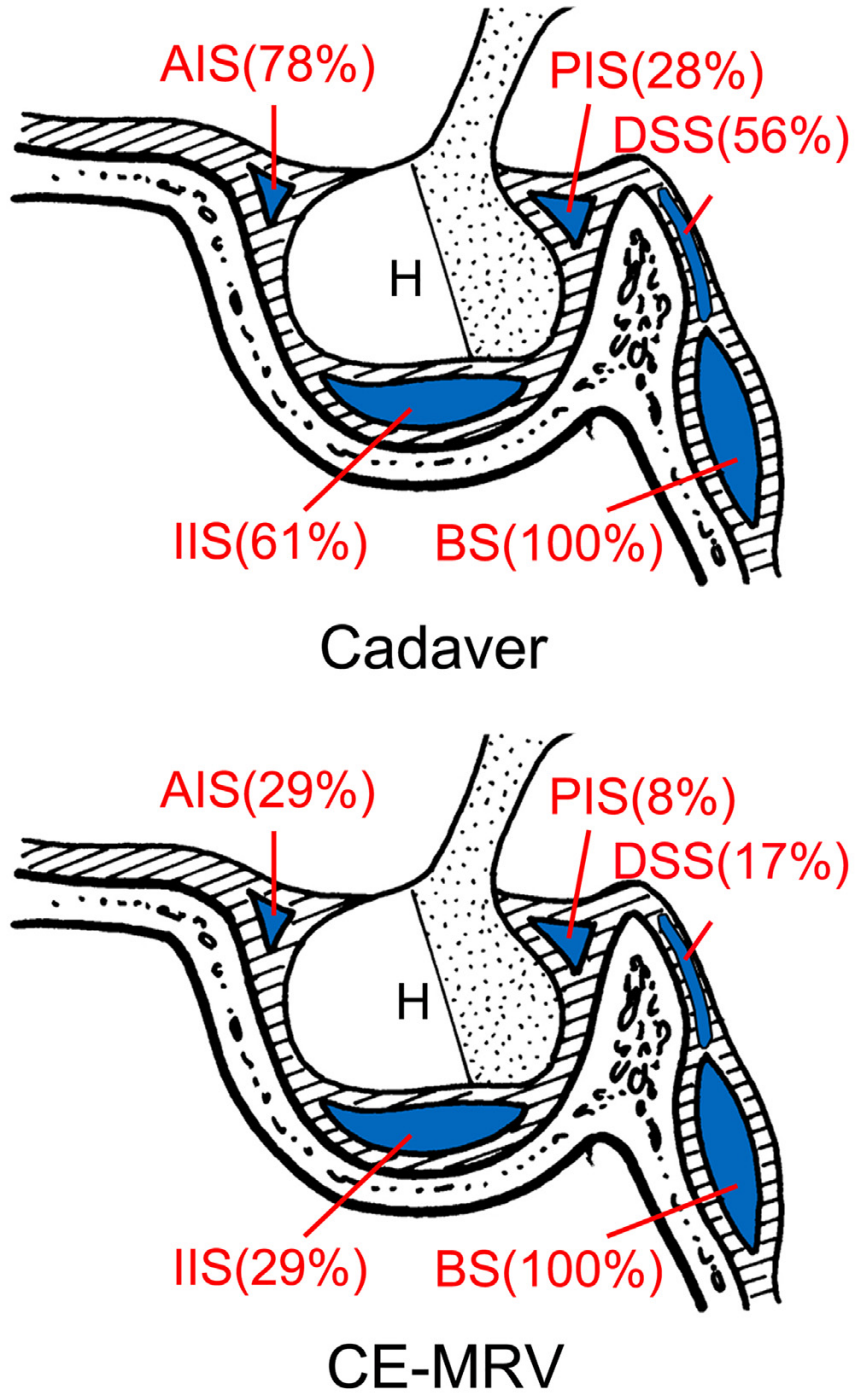
Illustration showing the rate of different intercavernous sinuses in cadaver and MRV. AIS: anterior intercavernous sinus; BS: basilar sinus; DSS: dorsum sellae sinus; H: hypophysis; IIS: inferior intercavernous sinus.

In cadavers, the AIS, IIS, PIS, DSS, and BS was found in 78%, 61%, 28%, 56%, and 100% of patients, respectively. In CE-MRV, the AIS, IIS, PIS, DSS, and BS was found in 29%, 29%, 8%, 17%, and 100% of patients, respectively (Table 1, Fig 5). Using the result obtained from the adult cadavers as a reference, the CE-MRV found 37% of AIS, 48% of IIS, 30% the PIS, 30% of DSS, and 100% of BS. Although there was no statistical difference between the supraoinferior diameter observed by cadavers and CE-MRV (Table 2), the average anteroposterior diameters of the intercavernous sinus measured in CE-MRV were larger than that in the cadavers (Table 3).

**Table 1:** The rate of intercavernous sinuses in cadaver and CE-MRV.

intercavernous sinus	Cadaver(N = 18)	CE-MRV(N = 24)	*P* value
anterior intercavernous sinus	14(78%)	7(29%)	0.001
inferior intercavernous sinus	11(61%)	7(29%)	0.039
posterior intercavernous sinus	5(28%)	2(8%)	0.031
basilar sinuses	10(56%)	4(17%)	0.007
dorsum sellae sinuses	18(100%)	24(100%)	1.000

**Table 2:** Supraoinferior diameters of the intercavernous sinuses in cadaver and CE-MRV (mm).

intercavernous sinus	Cadaver(N = 18)	CE-MRV(N = 24)	*P* value
anterior intercavernous sinus	1.99±1.01 (0.48~4.52)	2.21±0.60 (1.50~3.20)	0.599
inferior intercavernous sinus	1.11±0.68 (0.36~2.46)	1.51±0.40 (1.00~2.10)	0.176
posterior intercavernous sinus	1.15±0.42 (0.78~1.70)	1.40±0.42 (1.10~1.70)	0.503
dorsum sellae sinus	2.96±0.81 (1.50~4.60)	3.04±0.48 (2.60~3.70)	0.857
basilar sinuse	14.4±5.76 (4.20~24.9)	14.8±3.71 (7.10~26.6)	0.806

**Table 3:** Anteroposterior diameters of the intercavernous sinuses in cadaver and CE-MRV (mm).

intercavernous sinus	Cadaver(N = 18)	CE-MRV(N = 24)	*P* value
anterior intercavernous sinus	1.25±0.63 (0.60~2.66)	3.47±0.87 (2.80~5.40)	0.000
anterior intercavernous sinus	1.25±0.63 (0.60~2.66)	3.47±0.87 (2.80~5.40)	0.000
inferior intercavernous sinus	3.05±1.67(0.80~5.46)	5.31±1.37 (3.70~7.10)	0.009
posterior intercavernous sinus	0.99±0.30 (0.58~1.38)	3.20±0.85 (2.60~3.80)	0.002
dorsum sellae sinus	2.60±1.32 (1.28~4.98)	4.18±0.59 (3.60~5.00)	0.043
basilar sinuses	1.56±0.74 (0.40~3.00)	3.67±0.65 (2.40~5.60)	0.000

### Type of intercavernous sinus

**Fig 6:**
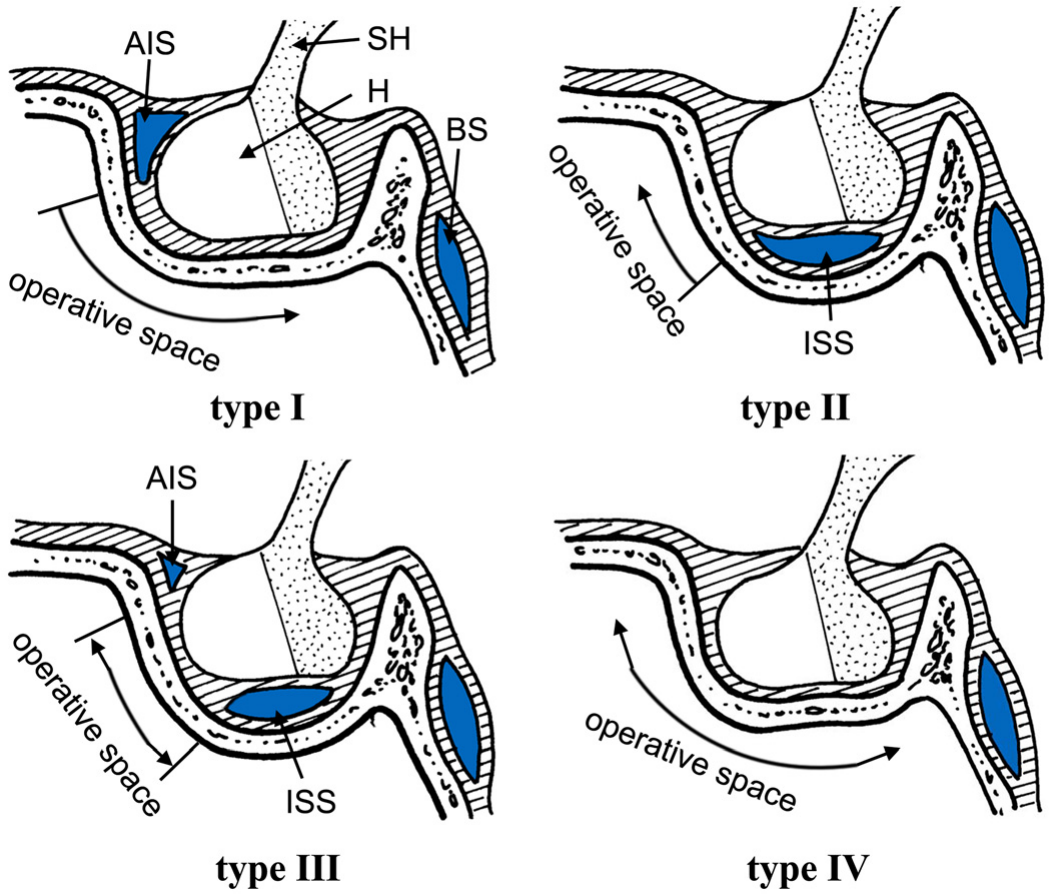
Illustration showing the operative space of sphenoid sinus in different types of intercavernous sinus. AIS: anterior intercavernous sinus; BS: basilar sinus; H: hypophysis; IIS: inferior intercavernous sinus; SH: stalk of hypophysis.

According to whether the AIS and IIS were present, four types of intercavernous sinuses were identified in cadavers and CE-MRV (Fig 6). **Type I:** the AIS but not the IIS was found in the cadavers or the patients (Fig 1). This type was found in 28% of cadavers and 21% of the CE-MRV. Enough operative space existed below the AIS. **Type II:** the IIS but not the AIS was found in the cadavers or the patients (Fig 2). This type was found in 11% of cadavers and 21% of the CE-MRV. Enough operative space existed above the IIS. **Type III:** the AIS and IIS were found simultaneously (Fig 3). This type was found in 50% of cadavers and 8% of the CE-MRV. Narrow operative space existed between the upper pole of the IIS and the lower pole of the AIS. **Type IV:** neither AIS nor the IIS were found (Fig 4). This type was found in 11% of cadavers and 50% of the CE-MRV. A large operative space existed for all of the pituitary fossa.

## Discussion

The pterional route, the subfrontal route and other transcranial approaches played a significant role in the traditional treatment of pituitary adenomas [23–26]. Because of the high mortality and morbidity and the widespread use of endoscope and diagnostic imaging, transsphenoidal surgery is generally considered by neurosurgeons to be the first-choice operative approach for pituitary adenoma [27–32]. The key factors ensuring the advantages of this approach include no obvious bleeding, clear visualization and complete exposure during operation [13]. Due to specific positions and anatomical variations, the the intercavernous sinus injury is the most common reason for the bleeding [2]. In the present anatomical study, different intercavernous sinuses have a high frequencies, from 28% to 100%. If the intercavernous sinuses were ignored by neurosurgeons during the operative process, bleeding may lead to poor visualization.

Whether the neuro-imagings can identify the intercavernous sinus has increasingly become a problem for the applicability of the operative approach. Catheterization of the inferior petrosal sinus has provided good visualization of the intercavernous sinus, and was used to diagnose the pituitary adenomas [17, 18]. This invasive examination restricted further use, and fewer researches have shown the ability of neuro-imaging to observe the intercavernous sinus in patients. With its exceptional soft-tissue resolution, MRI is becoming increasingly popular as a non-invasive means of evaluating the intracranial system. The pituitary gland is the most important site-specific organ for other structures in the sella turcica. In the CE-MRV, the entire gland enhanced homogeneously, and the intercavernous sinus had a higher intensity surrounding the gland. Neurosurgeons can make use of the gland as a coordinate for different intercavernous sinuses. In our study, different intercavernous sinuses were identified in all of the 24 patients.

Using the cadaver data as a reference, the CE-MRV found 37% of AIS, 48% of IIS, 30% the PIS, 30% of BS, and 100% of DSS. Although there was no statistical difference between the suprainferior diameter observed by cadavers and CE-MRV, the average anteroposterior diameters of the intercavernous sinuses measured in CE-MRV were larger than that in the cadavers. This supports the common belief that the size of a vein, rather than its drainage area, is important for depicting the vein on neuro-images [33, 34]. Different anatomical planes can be used to observe different intercavernous sinuses. The AIS was clearly shown in the transverse plane most of the time. However, the neuroradiologist could only identify the AIS and DS in the transverse plane. The IIS was clearly shown in the coronal plane most of the time, and again, the neuroradiologist could only identify the AIS and DS in this plane. All of the intercavernous sinuses can be showen in the median sagittal plane from different locations around the gland. In the multi-planar reconstruction imaging, the anatomical revelations based on the transverse and coronal plane can be compacted onto an image in the sagittal plane.

Except for the basilar sinus, the occurrence rate observed in CE-MRV was less than that of the cadaver. The following reasons may contribute to this. Firstly, the diameter of some intercavernous sinuses were less than 0.7 mm, which cannot be displayed by the MRV. Secondly, display of venous structures with the neuro-imagings is related to not only anatomical factors but also radiological factors that include the volume of contrast, iodine contain, injection speed, scanning time, TR/TE/FA sequence, blood flow, various pathological conditions of the patients, etc. Finally, sometimes the boundary between the DSS and BS was undefined, which lead to the occurrence rate of DSS in CE-MRV to be used only as a reference. To obtain a true specificity, neuro-imagings will need to be compared to the cadavers of the same people that have been imaged, which is not practical at all. Thus, extreme care needs to be taken when interpreting our comparison data. In order to improve the CE-MRV observation of the cavernous sinus, the sensitivity of the display and measurement is still needed in order to be studied further.

During the dural opening, the intercavernous sinus, especially the anterior and inferior intercavernous sinuses, are prone to bleeding under inappropriate manipulation [35]. According to whether the ASS and ISS were present, the intercavernous sinus was divided into four types. The four different types could be identified by the CE-MRV. Based on their types (Types I, II, III and IV), four operative plans should be used in the transsphenoidal surgical approach. (1) When the AIS exists by itself, the dural opening should be incised in the lower pole of the AIS. Subsequently, neurosurgery is used to achieve a wide operative field, which leads to a smaller chance of damage to the intercavernous sinus. (2) When the IIS exists by itself, the dural opening should be incised in the upper pole of the IIS. Neurosurgery can still achieve a wide operative yield, but should be carefully exercised in order to avoid injury to the IIS. (3) When the AIS and IIS exist simultaneously, the dural opening should be incised between the upper pole of the IIS and the lower pole of the AIS. Neurosurgery can only reach a small operative yield and it is difficult to treat the lesions. If there is a large AIS and a larger ISS, the neurosurgeon should select the transcranial surgerical approach in order to avoid heavy bleeding during the transsphenoidal surgery. (4) When the AIS and IIS do not exist simultaneously, the transsphenoidal surgery is relatively the most secure operative approach. In spite of the small intercavernous sinus, which was not shown by the CE-MRV but may actually exist within the patient, the neurosurgeon should treat the small bleeding during the operation using an electric coagulation procedure.

In conclusion, this study revealed that the morphology and classification of the intercavernous sinus can be identified by CE-MRV, especially for the larger vessel, which causes bleeding more easily. Therefore, CE-MRV provides a reliable measure for individualized preoperative planning during transsphenoidal surgery.

## Supporting Information

S1 File The relevant data about this manuscript. (XLS)
